# Evaluating the Completeness and Quality of Discharge Summaries: A Clinical Audit on Documentation Deficiencies and Pathways for Improvement

**DOI:** 10.1192/bjo.2026.11565

**Published:** 2026-06-30

**Authors:** Imtiaz Ahmad Dogar, Tayyib Tariq, IQRA AIN ALI, Fasiha Tahir, Habib Ur Rahman Yazdani

**Affiliations:** Faisalabad Medical University, Faisalabad, Pakistan

## Abstract

**Aims:**

Discharge summaries are essential documents that promote continuity of care and patient safety and communication between inpatient and outpatient providers. They are especially important in the psychiatric setting since they need to contain accurate records of the mental status of the patient, risk evaluation, and the education of both the patient and caregiver to avoid relapses and adverse events. This audit aims at evaluating the quality of psychiatric discharge summaries in our hospital and finding out the main areas that need improvement to be used in quality improvement initiatives

**Methods:**

This clinical audit was conducted to determine the completeness and quality of 80 discharge summaries of patients admitted to the Psychiatry Department of Allied-2 Hospital, Faisalabad, using a structured checklist based on Royal College of Physicians (RCP) guidelines, between January and June 2025. The main components were patient identifiers, diagnosis, mental state at discharge, risk assessment, treatment summary, medications, follow-up plan, counseling documentation, and legibility. The data analysis was done descriptively using frequencies and percentages, with 100 percent compliance considered the gold standard.

**Results:**

The degree of compliance with RCP standards was overall 47.5, which was significant. There were high scores in the elements of administrative data such as admissions and discharge dates (100), final psychiatric diagnosis (100), a review of treatment (100), discharge medications (100), and patient identifiers (98.75). Nevertheless, compliance among critical psychiatric-specific elements was very low: presenting complaint (0%), examination of mental state at discharge (0%), documented risk assessment (1.25%), patient and care giver counseling (1.25%), and typed/legible discharge summaries (0%). There were critical deficiencies in five components that had a great impact on patient safety, continuity of care, and managing risks.

**Conclusion:**

A review audit should be conducted to assess the effects of this intervention and to ensure that the RCP standards are followed so that psychiatric discharge paperwork and patient outcomes are improved. The absence of documentation of the mental state examination, risk assessment, and counseling places patients at a significant safety risk. To be able to deal with these issues, electronic discharge summaries and standardized templates with mandatory psychiatric assessments, structured counseling protocols, and targeted training of personnel should be introduced.

